# Metagenomic analysis of bile salt biotransformation in the human gut microbiome

**DOI:** 10.1186/s12864-019-5899-3

**Published:** 2019-06-25

**Authors:** Promi Das, Simonas Marcišauskas, Boyang Ji, Jens Nielsen

**Affiliations:** 1 Department of Biology and Biological Engineering, Chalmers University of Technology, SE-41296 Gothenburg, Sweden; 2 Novo Nordisk Foundation Center for Biosustainability, Technical University of Denmark, DK-2800 Lyngby, Denmark

**Keywords:** Secondary bile acids, Gut microbiota, IBD, Bioinformatics, Metagenomics, Metabolomics, And bile salt biotransformation genes

## Abstract

**Background:**

In the biochemical milieu of human colon, bile acids act as signaling mediators between the host and its gut microbiota. Biotransformation of primary to secondary bile acids have been known to be involved in the immune regulation of human physiology. Several 16S amplicon-based studies with inflammatory bowel disease (IBD) subjects were found to have an association with the level of fecal bile acids. However, a detailed investigation of all the bile salt biotransformation genes in the gut microbiome of healthy and IBD subjects has not been performed.

**Results:**

Here, we report a comprehensive analysis of the bile salt biotransformation genes and their distribution at the phyla level. Based on the analysis of shotgun metagenomes, we found that the IBD subjects harbored a significantly lower abundance of these genes compared to the healthy controls. Majority of these genes originated from Firmicutes in comparison to other phyla. From metabolomics data, we found that the IBD subjects were measured with a significantly low level of secondary bile acids and high levels of primary bile acids compared to that of the healthy controls.

**Conclusions:**

Our bioinformatics-driven approach of identifying bile salt biotransformation genes predicts the bile salt biotransformation potential in the gut microbiota of IBD subjects. The functional level of dysbiosis likely contributes to the variation in the bile acid pool. This study sets the stage to envisage potential solutions to modulate the gut microbiome with the objective to restore the bile acid pool in the gut.

**Electronic supplementary material:**

The online version of this article (10.1186/s12864-019-5899-3) contains supplementary material, which is available to authorized users.

## Background

Microbial modification of human-derived bile acids (BAs) is known as secondary BAs, which provide cross-talk between the human host and gut bacteria in the large intestine [1]. Primary BAs such as cholic acid (CA) and chenodeoxycholic acid (CDCA) that are produced in the liver are modified to deoxycholic acid (DCA) and lithocholic acid (LCA) in the colon respectively. These secondary BAs have been known to regulate several host metabolic processes via receptor signaling, including the farnesoid X receptor (FXR), the liver X receptor (LXR), the G-protein coupled receptor TGR5, and the vitamin D receptor present in the gut, the liver and in the periphery [2]. Concerning the anti-inflammatory and anti-microbial functions exerted by the secondary BAs, dysbiosis in the bile salt biotransformation potential of gut microbiota might influence the function of BAs in the host physiology.

Previous studies of small-sized cohorts or 16S amplicon based studies have identified significant differences in the fecal metabolites between the healthy and Inflammatory Bowel Disease (IBD) subjects [3–6]. From a growing body of evidence, two well-established IBD-associated taxonomic signatures have been observed: (i) phylum-level decrease in Firmicutes (especially of class Clostridia and family Lachnospiraceae), and (ii) phylum-level increase in Proteobacteria (especially of family Enterobacteriaceae) [7]. At metabolite level, mouse-models of inflammation were measured with a significant reduction of gut microbiota-derived bile acids [8–10]. This decrease has resulted in an altered pathophysiological response in the host via an altered-composition of the gut microbiome.

However, the extent of secondary BA metabolism by the gut microbiota remains less studied as the bacterial species that are specialized with bile acid metabolic genes have been characterized and studied in only few *Clostridium* species [11–14]. Furthermore, so far only a few of the enzymatic genes comprising the entire pathway of BA metabolism had been assessed from shotgun metagenomic sequences [15, 16]. None of the previous studies provided a complete picture considering all the relevant enzymatic genes, on the secondary bile acid metabolism, i.e., their prevalence in other bacterial species and abundances under different health states.

Henceforth, we sought to investigate secondary BA pathway-related protein and gene sequences based on sequence and structural domain conservation. Once they have been screened, estimation of these sequences from the shotgun metagenomes would allow predicting the bile acid metabolic potential in a microbial community. Based on this approach, the following research questions were formulated: (i) What is the distribution profile of bile salt biotransformation protein (BSBP) homologs at phyla level? (ii) How are these BSBP homologs prevalent at strain level? (iii) How conserved or divergent these homologous protein sequences are with respect to the phylum and enzymatic category? (iv) Does the abundance of bile salt biotransformation genes (BSBGs) vary among the fecal metagenomes of similar group and between different groups? and (v) Could parallel observations be deduced at metabolite level based on the findings from gene abundance estimation?

## Results

To provide a comprehensive view on gut microbial-mediated bile acid metabolism, the following analyses were performed: (i) Identification of BSBP homologs in microbial strains and their distribution at phyla level; (ii) Analysis of protein-sequence similarity network (PSSN) of identified homologs for each enzymatic function; (iii) Quantitative comparison of BSBG abundances between healthy controls and IBD subjects; (iv) Phylum-level quantitative comparison of BSBG abundances between healthy controls and IBD subjects, and (v) Quantitative comparison of fecal bile acid metabolites between healthy controls and IBD subjects.

### Identification and distribution of bile salt biotransformation protein homologs

To identify BSBP homologs, BSBP sequences from *Clostridium scindens* were deployed as reference query sequences (Additional file 1: Table S1). The reasons for considering this species as reference are: (i) limited experimental data (in testing the bacterial growth sensitivity to BAs) or lack in availability of BSBP or BSBG reference dataset (like an ARG database for antibiotic-resistance genes) [17], and (ii) extensive characterization of enzymatic genes comprising the secondary bile acid metabolic pathway (as shown to be encoded by *bai* operon) in the species *C. scindens* [13, 14, 18]. Through sensitivity-specificity analysis and user-defined methods of criteria to select the optimal parameters (Additional file 1: Table S2 and S4), it was ensured that the selected sequences were true positive homologs and rejected sequences that were false positive and true negative homologs. Therefore, based on protein sequence and domain conservation, a total of 10,613 protein homologs were identified. These counts of protein sequences were distributed based on the phyla and enzyme-homology (Table 1).

**Table 1:** Distribution of the total number of bile biotransformation protein homologs grouped by respective phylum

Phylum	BaiA	BaiB	BaiCD	BaiE	BaiF	BaiG	BaiH	BaiI	BaiJ	BaiK	BaiL	Bsh	HSD
Actinobacteria	86	317	25	538	146	475	67	0	15	68	44	36	182
Bacteroidetes	158	1	2	1	0	1	2	0	3	0	23	4	264
Firmicutes	385	13	435	20	96	244	424	2	130	92	576	889	409
Fusobacteria	5	0	2	0	0	0	2	0	44	0	0	0	11
Proteobacteria	114	152	92	104	460	73	85	0	31	534	84	2	598
Verrucomicrobia	1	0	0	1	0	0	0	0	0	0	2	0	2

**Fig. 1:**
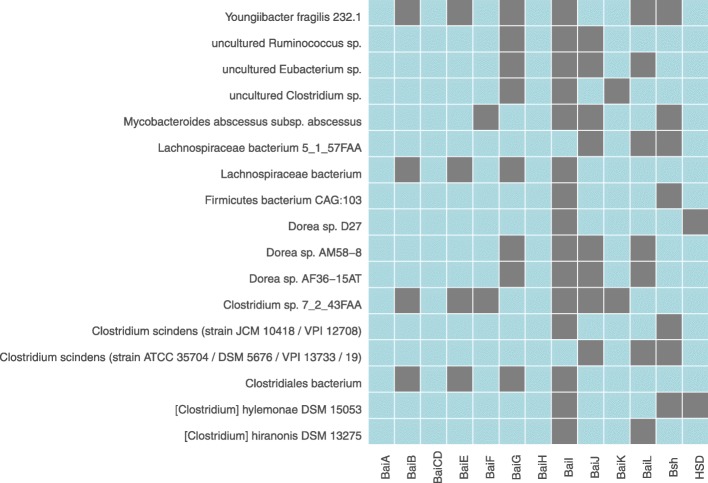
Prevalence of bile salt biotransformation protein homologs in bacterial strains, where at least more than half of the reference proteins were identified. The grey and blue color indicates the absence and presence of the protein homolog respectively

Based on the taxonomic origin (i.e., at strain level) of protein sequences, all the identified protein homologs were inspected for the presence and absence of each functional enzyme category as presented in Additional file 2: Table S1. Microbial strains that were prevalent for more than half of the enzyme categories were shown in Fig. 1. From the presence and absence heatmap (Fig. 1), we observed that species from the same genus had a similar profile pattern of BSBP homologs. Moreover, in a majority of the species, the distribution pattern of these enzymatic sequences was not as similar to that in the reference *bai* operon from *C. scindens* (Additional file 2: Table S1). Microbial strains of Lactobacillus species were known to deconjugate bile salts [19, 20], and we observed the same with prevalence for BSH homologs. However, enteric pathogens like Shigella, Salmonella, and Klebsiella were prevalent for Bai-like proteins but not for BSH homolog. (Additional file 2: Table S1).

### Protein-sequence similarity network of bile salt biotransformation protein homologs

**Fig. 2:**
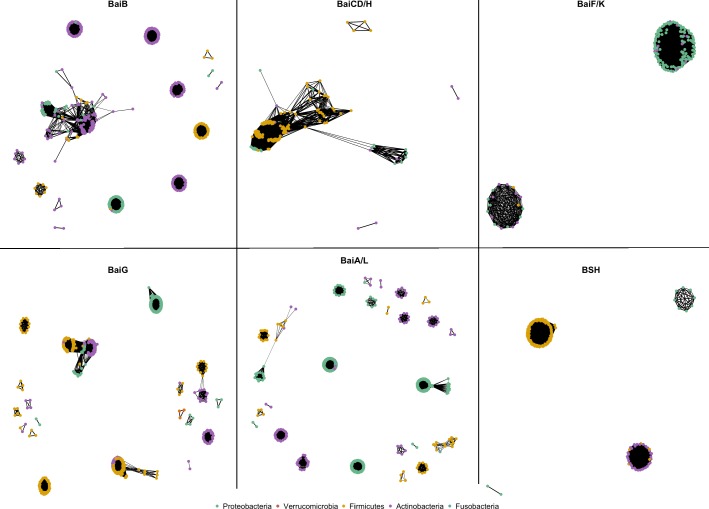
Construction of sequence-based protein similarity network of each bile salt biotransformation enzyme homolog, where each node represents a sequence, connected to another node as defined by an edge metric (which is optimal e-value and pairwise sequence identity of at least greater than 30%)

Next, to evaluate the genetic diversity of identified homologs at the phylum level, protein sequences from each enzyme category were analyzed using sequence-based similarity network (Fig. 2). For a multiple sequence alignment (for at least thousands of sequences), the low resolution of nodes is often encountered due to a low number of informative phylogenetic sites [21]. Hence, to overcome this challenge, we performed the following analysis. In a PSSN, the number of cluster formation would differ based on the threshold value, such as there would be fewer clusters for relaxed threshold value and more clusters for stringent threshold value. The optimal threshold value for each network shown in Fig. 2 is provided in Additional file 1: Table S4. From a broad perspective, we observed that sequences from similar phylum were co-localized on the network, with minor exceptions where sequences from different phylum were clustered together.

However, on a detailed level of inspection, BaiCD/H showed a single cluster even at higher stringent edge thresholds in comparison to the optimal values of other networks, which denotes less divergence within these homologous sequences across diverse phylum. While for the other networks, there was a variation in the number of clusters from a range of relaxed to stringent threshold criteria (Fig. 2 and Additional file 1: Table S4). For instance, a network for BaiG and BaiA/L (enzyme category with few protein connections or more clusters) identifies farther sequence divergence among the putative homologs. For each enzyme category, even though these protein homologs were identified through sequence conservation to a reference sequence, the clustering pattern reveals the biological degree of divergence or convergence among the identified homologs at their phyla-level.

### Comparative metagenomic analysis of bile salt biotransformation gene abundances between healthy and IBD subjects

Through investigation of gene abundances (for the identified list of protein homologs, as described in the Methods section) in the fecal metagenomes, bile salt biotransformation potential was compared quantitatively in the gut microbiota of healthy and IBD subjects.

**Fig. 3:**
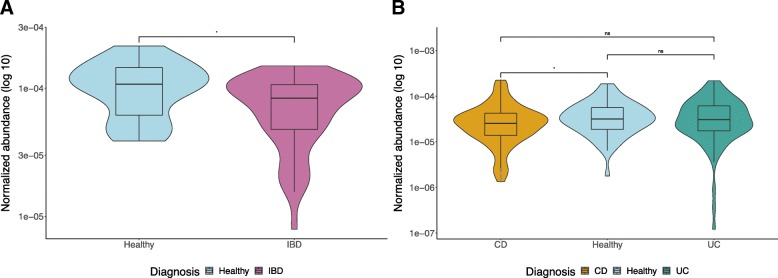
Quantitative comparison of normalized abundance of total BSBGs between healthy and IBD individuals in (**a**). Spanish cohort, and (**b**). American cohort. The shape refers to the kernel probability density of the data at different values. The boxplots inside the violin plot represent the interquartile range between the first and third quartiles with the median line inside the boxes, whereas the whiskers indicate the minimum and maximum values from the data distribution. The asterisks on the top indicate ns: *p* > 0.05, *: *p* < = 0.05, **: *p* < = 0.01, ***: *p* < = 0.001, ****: *p* < = 0.0001 (Mann-Whitney Wilcoxon test). IBD subjects diagnosed with subtype Crohn’s disease and Ulcerative colitis is abbreviated as CD and UC respectively

In the METAHIT-Spanish cohort, on comparing the normalized gene abundance of total BSBGs between healthy (*n* = 14) and IBD (*n* = 25) subjects, we found a significant difference between the abundance values of healthy and IBD subjects. The results in Fig. 3a show that the mean of the normalized abundance of BSBGs was lower in IBD subjects (i.e., 7.77e-05) than that of healthy controls (i.e., 1.08e-04) (Mann–Whitney–Wilcoxon test, *p* < 0.05), suggesting lower potency of BA biotransformation in the IBD subjects than that of the healthy controls. Our results here were consistent with findings from similar analyses performed in Labbe et al. [22], where samples from the MetaHIT cohort were also analyzed. However, further statistical comparisons between the subtypes of IBD subjects (i.e., Ulcerative colitis (UC) and Crohn’s disease (CD)) could not be performed due to an insufficient number of CD samples.

In the iHMP-American cohort, on comparing the normalized gene abundance of total BSBGs in the healthy (*n* = 18) and IBD (*n* = 65) subjects, we could not find any significant difference between the abundance values of healthy and IBD subjects. However, on all possible combination of statistical comparisons between the healthy, UC and CD subjects, the results in Fig. 3b show that the mean of normalized abundance of BSBGs was lower in the CD subjects (i.e., 3.68e-05) than that of the healthy controls (i.e., 4.34e-05) (Mann–Whitney–Wilcoxon test, *p* < 0.05), suggesting lower potency of BA biotransformation in the CD subjects than that of the healthy controls. No significant differences were observed between the following comparisons (i) healthy and UC subjects, and (ii) UC and CD subjects.

### Comparative metagenomic analysis of bile salt biotransformation gene abundances among healthy subjects from different countries

Similar to above, through investigation of gene abundances (for the identified list of protein homologs) in the fecal metagenomes, we compared the bile salt biotransformation potential in the gut microbiota of healthy subjects from three different countries, i.e., USA (*n* = 18), Denmark (*n* = 86) and Spain (*n* = 14). Additional file 1: Figure S2 revealed variation in the normalized abundances of the total BSBGs. From all possible combination of pairwise comparisons among different countries, we observed that the significant differences among healthy individuals of different countries. While individuals from the USA showed a significant difference from that of Denmark and Spain (Mann Whitney Wilcoxon, *p* < 0.01), no significant difference was found between the healthy individuals of Denmark and Spain. These findings hint at the possibility of diet as an important factor that could influence the composition of the gut microbiota, thereby leading to the differences in the abundance of BSBGs [23–25]. This analysis also highlights the importance of considering demographically matched samples as a prerequisite for comparative functional studies.

### Taxonomic-level comparative metagenomic analysis of bile salt biotransformation gene abundances between healthy and IBD subjects

**Fig. 4:**
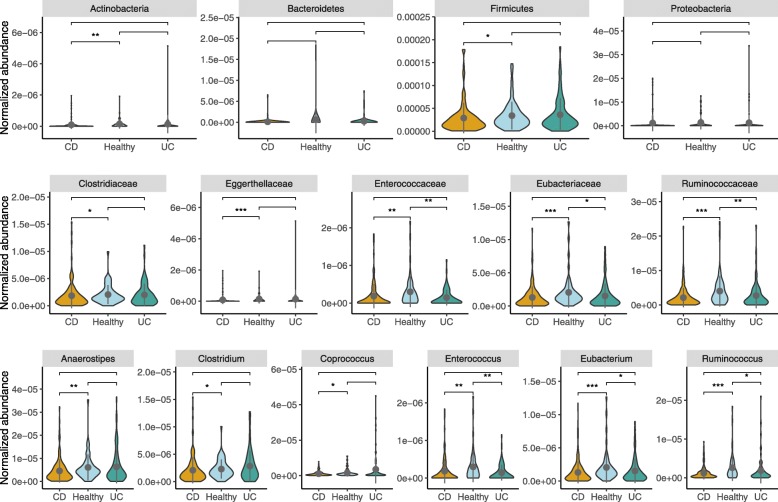
Quantitative comparison of normalized abundance of taxonomic lineage-specific BSBGs between healthy and IBD individuals in American cohort. The shape refers to the kernel probability density of the data at different values. The pointrange refers to the mean and error range value of the data distribution. IBD subjects diagnosed with subtype Crohn’s disease and Ulcerative colitis is abbreviated as CD and UC respectively

To evaluate the contribution of phyla accounting for BSBG abundances, each mapped gene was retrieved for its corresponding taxonomic lineage, and the normalized abundances of taxonomic-level BSBGs were computed accordingly for each group in both the cohort (Fig. 4). The results show that the genes from Firmicutes phylum accounted for the most considerable abundance for the BSBG in comparison to other phyla (Mann-Whitney-Wilcoxon test, *p* < 0.05). This observation, i.e., high abundance of genes from the Firmicutes phylum, is consistent with the existing literature, which states that species from Firmicutes phylum dominate in BA metabolism [16]. However, lower abundance of Firmicutes phylum-specific BSBGs in IBD subjects compared to that of healthy controls further confirms one of the typical IBD signature (i.e., marked by a decreased abundance of species from Firmicutes) (Mann-Whitney-Wilcoxon test, *p* < 0.05) [26–28]. Besides Firmicutes, BA genes from Actinobacteria were found to be lower in CD than in healthy controls (Mann-Whitney-Wilcoxon test, *p* < 0.05) (Fig. 4). Furthermore, no significant differences were observed in the level of BSBGs for the phyla: Bacteroidetes and Proteobacteria between the healthy and IBD subjects.

The family-level and genus-level analysis of bile salt biotransformation genes from Firmicutes phylum were investigated among CD, UC subjects, and healthy controls. As presented in Fig. 4, it is to be observed that genes from Enterococcaceae, Eubacteriaceae, and Ruminococcaceae were low in CD and UC subjects in comparison to healthy controls (Mann-Whitney-Wilcoxon test, *p* < 0.05). However, genes from Clostridiaceae and Eggerthellaceae, were found to be low in CD but not UC subjects in comparison to healthy controls (Mann-Whitney-Wilcoxon test, *p* < 0.05). Accordingly, genes from genera Enterococcus, Eubacterium, and Ruminococcus were found to be low in CD and UC subjects, while genes from genera Clostridium and Coprococcus were found to be low only in CD subjects than that of healthy controls (Mann-Whitney-Wilcoxon test, *p* < 0.05). Similar findings were obtained when performed in the other MetaHIT-Spanish IBD cohort (Additional file 1: Figure S3). These findings highlight the association of gene homologs from these genera to bile salt biotransformation potential in the context of IBD subjects, as supported in a study where genus-level abundance were correlated to bile acid levels in the feces of healthy donors [29].

### Comparative fecal metabolomics analysis of bile acids between healthy and IBD subjects

**Fig. 5:**
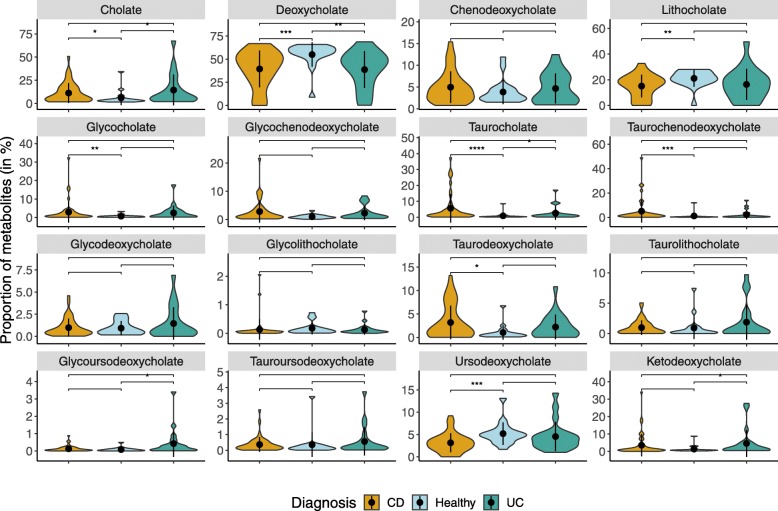
Quantitative comparison of bile acid metabolites between healthy and IBD subjects of American cohort. The shape refers to the kernel probability density of the data at different values. The pointrange refers to the mean and error range value of the data distribution. The asterisks on the top indicate ns: *p* > 0.05, *: *p* < = 0.05, **: *p* < = 0.01, ***: *p* < = 0.001, ****: *p* < = 0.0001 (Mann-Whitney Wilcoxon test). IBD subjects diagnosed with subtype Crohn’s disease and Ulcerative colitis is abbreviated as CD and UC respectively

To verify if the IBD individuals have reduced levels of secondary BAs corresponding to their predicted low abundance of BSBGs than that of healthy controls, metabolomics data from the iHMP cohort were analyzed. Fecal bile acid metabolites were measured in LC-MS (C18 negative ion mode analysis) [30, 31]. To compare the bile acid profile between healthy and subtypes (CD and UC) of IBD subjects, quantification of BAs was expressed in proportion, where the level of each BA was calculated to the total level of BAs. Figure 5 shows the proportion of a wide range of primary and secondary BAs, both in conjugated and unconjugated forms. The major BAs, cholate (primary BA) and deoxycholate and lithocholate (secondary BAs), were found to be in high and low levels in both the subtypes of IBD subjects than that of healthy controls (Mann-Whitney-Wilcoxon test, *p* < 0.05). The conjugated forms of primary BAs such as glycocholate, taurocholate, and taurochenodeoxycholate were found to be in high levels than healthy controls (Mann-Whitney-Wilcoxon test, *p* < 0.05), which suggests the lower abundance of genes encoding bile salt hydrolase [32, 33].

Furthermore, it was observed that there was a decrease in the levels of secondary BAs in CD than that of healthy controls. This decrease in the level of secondary BAs compared to the increased level of primary BAs once again suggests a decrease in the potency of BA biotransformation, which is consistent with our comparative metagenomic analysis. These results were in line with a similar analysis performed on this cohort [30, 34]. Altogether, the proportions of different forms of conjugated and primary BAs were found at higher levels in IBD subjects than that of healthy controls. These observations were congruent to that found in Duboc et al. [35, 36] where low levels of secondary BAs were found in IBD subjects, who were in the active phase and clinical remission state.

## Discussion

In this study, we performed bioinformatic analysis on secondary bile acid metabolism and explored their abundances in the whole-genome metagenomic sequences of healthy and IBD subjects. The overall aim of this work was to gain an understanding of the bile salt biotransformation potential of the human gut microbiota. This provided insights into how the change in the abundance of BSBGs altered the level of BA metabolic profiles in a dysbiotic state.

Based on the analyses that have been performed, we highlight the following results resolving back to our formulated objectives (i) BSBP homologs were prevalent across abundant phyla, such as Bacteroidetes, Firmicutes, Actinobacteria, and Proteobacteria. However, phyla- and genera-level differential gene abundance analysis revealed dominant role of Firmicutes and its associated genera for BSBGs; (ii) The prevalence of BSBP profile varied from a low to a high scale of spectrum; (iii) PSSN revealed the degree of divergence for different enzymatic categories; (iv) BSBG-specific quantitative estimation of shotgun metagenomes suggests lower potency of bile salt biotransformation in the gut microbiota of IBD subjects; (v) Evaluation at metabolomics level revealed parallel reduction of gut microbial-derived bile acids in IBD subjects compared to healthy controls.

Homology-based detection of BSBP homologs suggests three patterns as discussed: firstly, the presence of a partial set of homologs in comparison to the whole set of enzymatic categories might suggest a similar situation as found in *C. hiradonis* and *C. sordellii.* For instance, *C. hiradonis* carried *bai*BCDEA2FGHJ while *C. sordellii* carried only *bai*CDA2HE [37]. Alternatively, it could be a possibility of similar function with similar substrates to that of bile acid metabolism. Secondly, experimental approaches to elucidate the requirement for minimum *bai* gene set could clear the specific distribution of these genes across gut species. Thirdly, it is to be established which combination of Bai and BSH protein contribute to the phenotype of resistance or sensitivity to BAs, because, only a subset of species was found to carry BSH homologs, while other species lacked in BSH homolog. Extracellular enzymatic activities similar to BSH could perhaps provide functional compensation for species lacking the enzyme [38, 39].

Besides evaluation of BSBGs in IBD subjects, similar dysbiosis has been observed in cirrhotic patients, where a low-level input of primary BAs to the gut leads to a reduction of BA metabolizers in the large intestine. Accordingly, reduced levels of secondary BAs in the feces and increased levels of primary BAs were found in the serum and feces of cirrhotic patients [40–42]. As both the cases converge to a similar metabolic phenotype, further investigation at advanced level could avoid misclassification of these subjects. Contrarily, high physiological levels of secondary BA have been associated with colorectal cancer patients [43]. Due to their detergent-like properties, chronic exposure to higher concentration of BAs can damage cell membrane and induce pro-inflammatory pathways resulting in activation of ROS and genomic instability of colonic cellular DNA [44–48]. However, as IBD comprises of heterogeneous population of subtypes, inter-individual variation of gut microbiota and their expression potential among similar population further complicates the generalization and validation of bile acids needed to restore the bile acid pool across IBD cohorts [49]. Hence, any deviation from the physiological range of secondary BAs could contribute to a disease phenotype.

The biological relevance of these secondary and tertiary BAs, such as DCA, LCA, ursodeoxycholic acid (UCDA) have known to be potent agonists for several BA receptors and exert anti-inflammatory effects in the colon. For instance, they bind to TGR5 and inhibit the production of cytokines once encountered with lipopolysaccharides from gram-negative bacteria [50]. LCA could also regulate adaptive immune responses via VDR signaling [51] whereas FXR activation maintains the production of BAs and their homeostasis [52]. These properties of secondary BAs suggest their beneficial role in reducing inflammation in IBD [53]. The importance of bile acid therapy has been shown in animal studies and in human-trials [54–58]. Of particular interest, UDCA, which is an FDA-approved drug for cholestatic liver disease, has been shown to ameliorate inflammation in dextran sulfate sodium-induced colitis mice [59].

Considering the relation between BA metabolism and IBD, one potential application using probiotics has alleviated disease remission of relapsing UC patients [60, 61]. To tolerate bile acid stress in the gut, the ability to hydrolyze bile salts is often considered for probiotic strain selection [20, 62]. Therefore, to improve the gut health of IBD subjects, had they been supplemented with BSH-active strain formulated in probiotics along with their routine anti-inflammatory drug, synergistic effects of this combination could help in the conversion of bile salts to secondary BAs. This approach has improved the clinical features of relapsing UC patients suggesting that interventions targeting BA metabolism may have therapeutic implications [60, 61]. Reconstitution of gut microbiota with BSH-active species through fecal microbiota transplantation has been effective in prevention of recurrent *Clostridium difficle* infection [36]. Overall, this study further stresses that IBD-associated altered BA biotransformation could remove anti-inflammatory effects of BAs and the dysbiotic state could participate in the chronic inflammatory loop tipping towards an immunosuppressed state of health.

## Conclusions

Taken together, our approach of targeted identification and quantification of bile salt biotransformation has enabled us to realize the impact of gut microbial mediated metabolic effects on the host system. This functional dysbiosis was observed in the gut microbiota of IBD individuals, who were in early-onset IBD or clinical remission stage. We also observed that the reduction of Firmicutes-specific bile acid-related genes is in line with the compositional reduction of Firmicutes (especially species with bile metabolic potential). From a broad view of the omics lens, the metagenomic predictions and the metabolomics evaluations were consistent in both approaches. However, verification of identified homologs from few of the cultivable species, through a reductionist approach, could further verify the biological significance of sequence conservation. Nonetheless, gut microbial composition and their function could potentially tip a compensated state of immune balance in favor of chronic disease in IBD hosts.

## Methods

### IBD cohort description

Shotgun metagenomes were obtained from European Nucleotide Archive at EMBL-EBI under the accession number PRJEB2054 and PRJNA389280.

From PRJEB2054, 14, 4, and 21 samples of Healthy (H), Crohn’s disease (CD), and Ulcerative colitis (UC) respectively were analyzed for differential BSBG abundance analysis [63]. Both subtypes of IBD subjects were in clinical remission for at least 3 months and had stable maintenance therapy with mesalazine or azathioprine. Individuals who were undergoing antibiotic treatment for at least 4 weeks before fecal sample collection were excluded [64].

From PRJNA389280, 18, 44, and 21 samples of Healthy (H), Crohn’s disease (CD) and Ulcerative colitis (UC) respectively were analyzed for differential BSBG abundance and BA metabolite analysis. These samples had paired or coordinated metagenomics and metabolomics data available. None of these IBD subjects had a history of terminal ileal resection. Detailed information for the sample selection criteria on IBD subjects is available in the original article [30].

### Bioinformatic identification of bile salt biotransformation protein homologs

To identify BSBP homologs from the UniProt database [65], experimentally verified BSBPs from *Clostridium scindens* (strain JCM 10418 / VPI 12708) and other species were deployed as query sequences (Additional file 1: Table S1 and Figure S1). The selection process of BSBP homologs was performed in two sections. First, screening the candidate BSBPs using BLASTp [66] under optimal threshold parameters (Additional file 1: Table S2). To determine the threshold for BLASTp search, protein sequences with known BSB function and non-BSB function were used as test data. For instance, BSB proteins from *Eggerthella lenta* was used as positive control [67, 68], while protein sequences from Helicobacter, Prevotella, and Porphyromonas were used as negative control [69–74]. The accuracy of BLASTp was estimated at different similarity thresholds with defined e-value and coverage (Additional file 1: Table S2).

Using the BSH-specific HMM and PVA-specific HMM [75, 76], protein sequences of bile salt hydrolase (BSH) category were identified to make a distinct classification from penicillin-v-acylase (PVA) enzyme sequences, as both shared identical domain CBAH superfamily. Sequences that had higher e-value in the BSH cluster compared to that in the PVA cluster were selected for further analysis.

Secondly, the candidate BSBP homologs were searched against the Pfam 31.0 database to identify their functional domains using the default GA (gathering cut-offs) [77]. Sequences with identical domain organizations were retained for the following analysis (Additional file 1: Table S3). Additionally, homologous protein sequences that were filtered across the Pfam domain analysis were checked for their functional annotation using the eggNOG algorithm [78] with DIAMOND as mapping mode and other default parameters. Sequences that mapped to similar annotations to the reference protein were considered for the final reference database. The taxonomic lineage of the identified gene sequences was retrieved from the UniProt website (dated on October 2018) [65].

### Construction of protein sequence similarity network (PSSN)

An all-by-all protein BLAST was performed for all the homologous sequences to be analyzed in the dataset. Pairs of sequences with significant similarity were selected through the application of a series of pairwise protein BLAST e-value thresholds, where their distribution was used to define the optimal e-value cut-off. Subsequently, significant alignments (with e-value less than the optimal e-value and minimum of 30% pairwise similarity) were used to construct the PSSN (Additional file 1: Table S4). Each node of the network indicates a protein sequence, and the edge represents the relatedness between two nodes sharing significant similarity as defined by the edge metric (i.e., e-value and percentage identity). Networks were visualized with the ggnetwork package in R [79, 80].

### Functional metagenomic mapping

For quality assessment, publicly available fastq reads were subjected to a quality check using FastQC [81] with a minimum Phred score of 30. Each pair of fastq files was repaired to remove the singletons (if needed) using the script available at (https://github.com/BioInfoTools/BBMap/blob/master/sh/repair.sh). Bile salt biotransformation gene abundances were calculated using FMAP [82] with the default parameters. Gene sequences that were binned into the functional category of Kyoto Encyclopedia of Genes and Genomes (KEGG) pathway ko00121 were determined by mapping of UniRef to KEGG Orthologies (KOs) database [83, 84]. Then, the numbers of mapped reads were normalized to the total number of paired reads in the metagenomes. The taxonomic lineage for each mapped protein gene ID sequence was obtained from UniProt database [85].

### Statistical analysis

All the statistical analysis and graphics were performed using R version 3.5.0 [86]. In each cohort, samples were country-matched to avoid cross country-effects. Normality of the given dataset was assessed through the Shapiro–Wilk test. Differential abundance analysis between healthy and IBD subjects was tested using Mann-Whitney-Wilcoxon tests (for comparison between two groups), and Kruskal Wallis tests (for comparison among three groups). Multivariable linear regression was performed for age, BMI and gender on the gene abundance to identify confounding variable (*p* < 0.01) by unadjusted analysis.

## Additional files

Additional file 1: **Table S1.** List of query proteins and their basic description used as reference sequences to identify bile acid metabolic proteins (sourced from UniProt). **Table S2.** Optimal cut-off parameters chosen to filter the significant sequence hits were determined based on the distribution of BLAST pairwise percentage identity, e-value and query and target coverage. **Table S3.** List of query proteins with the description of their Pfam domain. **Table S4.** Optimal cut-off parameters chosen for the construction of protein sequence-based similarity network were determined based on the distribution of BLAST pairwise percentage identity and e-value values. **Figure S1.** Schematic representation of a generalized bile salt biotransformation pathway. Enzymatic proteins that were studied have been highlighted in red color. **Figure S2.** The normalized abundance of total BSBGs in healthy individuals sampled from the USA, Denmark, and Spain. The Y-axis of the boxplot refers to the normalized abundance, whereas the X-axis refers to the country of these healthy control groups. The shape refers to the kernel probability density of the data at different values. The asterisks on the top indicate ns: p > 0.05, *: p < = 0.05, **: p < = 0.01, ***: p < = 0.001, ****: p < = 0.0001 (Mann-Whitney Wilcoxon test). **Figure S3.** The normalized abundance of taxonomic-lineage specific BSBGs in healthy and IBD subjects sampled from Spain. The Y-axis of the violin plot refers to the normalized abundance, whereas the X-axis refers to the diagnosis. The shape refers to the kernel probability density of the data at different values. The pointrange refers to the mean and error range value of the data distribution. The asterisks on the top indicate ns: p > 0.05, *: p < = 0.05, **: p < = 0.01, ***: p < = 0.001, ****: p < = 0.0001 (Mann-Whitney Wilcoxon test). (DOCX 413 kb)

Additional file 2: **Table S1.** Prevalence of bile salt biotransformation protein homologs in bacterial strains, where numeric one and zero refers to the presence and absence of the homolog. (XLSX 335 kb)

## Data Availability

All the relevant identifiers and other related information employed in this work have been provided in the additional files. Analyzed metagenomics datasets were obtained from https://www.ebi.ac.uk/ena/data/view/PRJEB2054 and https://www.ebi.ac.uk/ena/data/view/PRJNA389280.

## Abbreviations

BSBG: Bile salt biotransformation gene
BSBP: Bile salt biotransformation protein
CD: Crohn’ disease
IBD: Inflammatory Bowel Disease
KEGG: Kyoto Encyclopedia of Genes and Genomes
KOs: KEGG Orthologies
PSSN: Protein sequence similarity network
UC: Ulcerative Colitis
