# Supplementary material for: Metagenomic analysis of bile salt biotransformation in the human gut microbiome
Source: BMC Genomics. 2019 Jun 25;20:517. doi: 10.1186/s12864-019-5899-3 (PMC6591925; doi:10.1186/s12864-019-5899-3)
Supplement: Supplementary file 1 — Table S1. List of query proteins and their basic description used as reference sequences to identify bile acid metabolic proteins (sourced from UniProt). Table S2. Optimal cut-off parameters chosen to filter the significant sequence hits were determined based on the distribution of BLAST pairwise percentage identity, e-value and query and target coverage. Table S3. List of query proteins with the description of their Pfam domain. Table S4. Optimal cut-off parameters chosen for the construction of protein sequence-based similarity network were determined based on the distribution of BLAST pairwise percentage identity and e-value values. Figure S1. Schematic representation of a generalized bile salt biotransformation pathway. Enzymatic proteins that were studied have been highlighted in red color. Figure S2. The normalized abundance of total BSBGs in healthy individuals sampled from the USA, Denmark, and Spain. The Y-axis of the boxplot refers to the normalized abundance, whereas the X-axis refers to the country of these healthy control groups. The shape refers to the kernel probability density of the data at different values. The asterisks on the top indicate ns: p > 0.05, *: p < = 0.05, **: p < = 0.01, ***: p < = 0.001, ****: p < = 0.0001 (Mann-Whitney Wilcoxon test). Figure S3. The normalized abundance of taxonomic-lineage specific BSBGs in healthy and IBD subjects sampled from Spain. The Y-axis of the violin plot refers to the normalized abundance, whereas the X-axis refers to the diagnosis. The shape refers to the kernel probability density of the data at different values. The pointrange refers to the mean and error range value of the data distribution. The asterisks on the top indicate ns: p > 0.05, *: p < = 0.05, **: p < = 0.01, ***: p < = 0.001, ****: p < = 0.0001 (Mann-Whitney Wilcoxon test). (DOCX 413 kb) [file 12864_2019_5899_MOESM1_ESM.docx]

**Table S1. List of query proteins and their basic description used as reference sequences to identify bile acid metabolic proteins (sourced from UniProt).**

| **Name** | **UniProt ID** | **Function** | **Length** | **Source** |
| --- | --- | --- | --- | --- |
| BaiA1 | P07914 | 3 alpha-hydroxy bile acid-CoA-ester 3-dehydrogenase 1/3 | 249 | *Clostridium scindens* (strain JCM 10418 / VPI 12708) |
| BaiA2 | P19337 | 3alpha-hydroxy bile acid-CoA-ester 3-dehydrogenase 2 | 249 | *Clostridium scindens* (strain JCM 10418 / VPI 12708) |
| HDHA | P0AET8 | 7-alpha-hydroxysteroid dehydrogenase | 255 | *Escherichia coli* (strain K12) |
| HDHA | P50200 | NADP-dependent 7-alpha-hydroxysteroid dehydrogenase | 267 | *Paeniclostridium sordellii* |
| HDHA | P0AET9 | 7-alpha-hydroxysteroid dehydrogenase | 255 | *Escherichia coli* O157:H7 |
| BaiB | P19409 | Bile acid- coenzyme A ligase | 520 | *Clostridium scindens* (strain JCM 10418 / VPI 12708) |
| BaiCD | P19410 | NAD+-dependent 7alpha-hydroxy-3-oxo bile acid-CoA-ester 4-dehydrogenase | 639 | *Clostridium scindens* (strain JCM 10418 / VPI 12708) |
| BaiE | P19412 | Bile acid 7-alpha dehydratase | 166 | *Clostridium scindens* (strain JCM 10418 / VPI 12708) |
| BaiF | P19413 | Bile acid-CoA transferase | 425 | *Clostridium scindens* (strain JCM 10418 / VPI 12708) |
| BaiG | P32369 | Bile acid transporter | 477 | *Clostridium scindens* (strain JCM 10418 / VPI 12708) |
| BaiH | P32370 | NAD+-dependent 7beta-hydroxy-3-oxo bile acid-CoA-ester 4-dehydrogenase | 661 | *Clostridium scindens* (strain JCM 10418 / VPI 12708) |
| BaiI | B4YSU5 | Bile acid-inducible operon protein I | 117 | *Clostridium hylemonae* |
| BaiJ | WP_025644103* | FAD-binding protein | 573 | *Clostridium scindens* (strain JCM 10418 / VPI 12708) |
| BaiK | ACF20979* | Bile acid-CoA transferase | 437 | *Clostridium scindens* (strain JCM 10418 / VPI 12708) |
| BaiL | ACF20980* | SDR family NAD(P)-dependent oxidoreductase | 243 | *Clostridium scindens* (strain JCM 10418 / VPI 12708) |
| BSH | P54965 | Choloylglycine hydrolase | 328 | *Clostridium perfringens* (strain 13 / Type A) |
| BSH | Q06115 | Choloylglycine hydrolase | 323 | *Lactobacillus plantarum* (strain ATCC BAA-793 / NCIMB 8826 / WCFS1) |

* denotes GenBank ID

**Fig S1. Schematic representation of a generalized bile acid biotransformation pathway. Enzymatic proteins that were studied have been highlighted in red color. Co-substrates and by-products have been highlighted in black and green color respectively. Conjugated primary bile salts are first deconjugated to primary bile acids by BSH enzyme. Then they enter the cell via BaiG membrane transporter. The primary bile acid gets transformed to secondary bile acid through series of reactions. The transporter required to export the secondary bile acid outside of the cell has not been determined.**

**Table S2. Optimal cut-off parameters chosen to filter the significant sequence hits were determined based on the distribution of BLAST pairwise percentage identity, e-value and query and target coverage.**

| **S.no** | **Name** | **Percentage Identity** | **E-value** | **Query Coverage** | **Target coverage** | **Accuracy** |
| --- | --- | --- | --- | --- | --- | --- |
| 1 | BaiA  HDHA  BaiL | 30 | 1e-10 | 80 | 80 | 93.33 |
| 2 | BaiB | 30 | 1e-10 | 75 | 75 | 96.67 |
| 3 | BaiCD  BaiH | 30 | 1e-10 | 80 | 80 | 96.55 |
| 4 | BaiF  BaiK | 30 | 1e-10 | 70 | 70 | 96.67 |
| 5 | BaiG | 30 | 1e-10 | 75 | 75 | 96.43 |
| 6 | BaiJ | 30 | 1e-5 | 70 | 70 | 100 |

**Table S3. List of query proteins with the description of their Pfam domain.**

| **Name** | **UniProt ID** | **Length** | **Alignment Start** | **Alignment End** | **HMM Accession** |
| --- | --- | --- | --- | --- | --- |
| BaiA1 | P07914 | 249 | 7 | 198 | PF00106.24 |
| BaiA2 | P19337 | 249 | 15 | 245 | PF13561.5 |
| HDHA | P0AET8 | 255 | 18 | 249 | PF13561.5 |
| HDHA | P50200 | 267 | 14 | 251 | PF13561.5 |
| HDHA | P0AET9 | 255 | 18 | 249 | PF13561.5 |
| BaiB | P19409 | 520 | 39  432 | 423  505 | PF00501.27  PF13193.5 |
| BaiCD | P19410 | 639 | 6  373 | 327  599 | PF00724.19  PF07992.13 |
| BaiE | P19412 | 166 | 15 | 140 | PF13577.5 |
| BaiF | P19413 | 425 | 13 | 377 | PF02515.16 |
| BaiG | P32369 | 477 | 3  149 | 130  349 | PF07690.15 |
| BaiH | P32370 | 661 | 8  384 | 337  625 | PF00724.19  PF07992.13 |
| BaiI | B4YSU5 | 117 | 39 | 155 | PF13577.5 |
| BaiJ | WP_025644103* | 573 | 122 | 553 | PF00890.23 |
| BaiK | ACF20979* | 437 | 15 | 380 | PF02515.16 |
| BaiL | ACF20980* | 243 | 2 | 240 | PF13561.5 |
| BSH | P54965 | 328 | 2 | 317 | PF02275.17 |
| BSH | Q06115 | 323 | 2 | 312 | PF02275.17 |

* denotes GenBank ID

**Table S4. Optimal cut-off parameters chosen for the construction of protein sequence-based similarity network were determined based on the distribution of BLAST pairwise percentage identity and e-value values.**

| **S.no** | **Name** | **Minimum percentage Identity** | **E-value** |
| --- | --- | --- | --- |
| 1 | BaiA  HDHA  BaiL | 30 | 1e-60 |
| 2 | BaiB | 30 | 1e-80 |
| 3 | BaiCD  BaiH | 30 | 1e-100 |
| 4 | BaiF  BaiK | 30 | 1e-70 |
| 5 | BaiG | 30 | 1e-75 |
| 6 | Bsh | 30 | 1e-80 |

**Fig S2. The normalized abundance (in log 10 scale) of total BSBGs in healthy individuals sampled from the USA, Denmark and Spain. The Y-axis of the boxplot refers to the normalized abundance, whereas the X-axis refers to the country of these healthy control groups. The shape refers to the kernel probability density of the data at different values The asterisks on the top indicate ns: p > 0.05, *: p <= 0.05, **: p <= 0.01, ***: p <= 0.001, ****: p <= 0.0001 (Mann-Whitney Wilcoxon test).**

**Fig S3. The normalized abundance of taxonomic-lineage specific BSBGs in healthy and IBD subjects sampled from Spain. The Y-axis of the boxplot refers to the normalized abundance, whereas the X-axis refers to the diagnosis. The shape refers to the kernel probability density of the data at different values. The asterisks on the top indicate ns: p > 0.05, *: p <= 0.05, **: p <= 0.01, ***: p <= 0.001, ****: p <= 0.0001 (Mann-Whitney Wilcoxon test).**
